# Femoral access in 100 consecutive subarachnoid hemorrhage patients: the "craniotomy" of endovascular neurosurgery

**DOI:** 10.1186/1756-0500-3-285

**Published:** 2010-11-05

**Authors:** Alexandra R Paul, Geoffrey P Colby, Martin G Radvany, Judy Huang, Rafael J Tamargo, Alexander L Coon

**Affiliations:** 1 Department of Neurosurgery, The Johns Hopkins Hospital, Baltimore, MD, USA; 2 Department of Radiology, The Johns Hopkins Hospital, Baltimore, MD USA

## Abstract

**Background:**

Femoral access is a fundamental element of catheter-based cerebral angiography. Knowledge of location of the common femoral artery (CFA) bifurcation is important as the risk of retroperitoneal bleeding is increased if the puncture is superior to the inguinal ligament and there is an increased risk of thrombosis and arteriovenous fistula formation if the puncture is distal into branch vessels. We sought to characterize the location of the CFA bifurcation along with the presence of significant atherosclerosis or iliac tortuosity in a contemporary series of subarachnoid hemorrhage (SAH) patients.

**Findings:**

The records of a prospective single-center aneurysm database were reviewed to identify 100 consecutive SAH patients. Using an oblique femoral arteriogram, the presence of significant atherosclerosis, iliac tortuosity, and the CFA bifurcation were assessed. The CFA bifurcation was graded according to its position with respect to the femoral head: below (grade 1), lower half (grade 2), and above the upper half (grade 3).

We found a CFA bifurcation grade 1 in 50 patients (50%, mean age 51.2 years), grade 2 in 40 patients (40%, mean age 55.5 years), and grade 3 in 10 patients (10%, mean age 58.2 years). Whereas 30 of 90 patients with CFA grades I or II were male (33%), only 10% with grade 3 were male (1 of 10, p = 0.12). Mean age for significant atherosclerosis was 65.5 +/- 2.6 years versus 50.9 +/- 1.6 years (p < 0.001) without, and iliac tortuosity was 64.9 +/- 2.4 years versus 50.3 +/- 1.6 years (p < 0.001) without.

**Conclusions:**

Although a requisite element of endovascular treatment in SAH patients, femoral access can be complicated by a high common femoral artery bifurcation and the presence of atherosclerotic disease and/or iliac artery tortuosity. In this study, we found a grade 3 (above the femoral head) CFA bifurcation in 10% patients, with 90% of these patients being female. We also found the presence of atherosclerotic disease and iliac tortuosity to be significantly more likely in patients older than 65 years of age.

## Background

Femoral access is a fundamental element of catheter-based cerebral angiography and neurointerventional procedures. The femoral artery is most commonly used for arterial access. The common femoral artery (CFA) is the continuation of the external iliac artery from the level of the inguinal ligament to its bifurcation into the profunda femoris (deep femoral artery) and the superficial femoral artery. The CFA is considered to be the safest site for arterial puncture however there is little published data relating the CFA and its bifurcation to the landmarks typically used to guide arterial puncture. Knowledge of these landmarks is important as the risks of retroperitoneal bleeding complications are increased if the puncture is superior to the inguinal ligament and there is an increased risk of thrombosis and arteriovenous fistula formation if the puncture is distal into branch vessels. It is commonly accepted that the CFA lies over the femoral head but the precise location of the CFA bifurcation is typically unknown [1,2]. We sought to characterize the location of the CFA bifurcation in patients presenting with subarachnoid hemorrhage (SAH). We also assessed the presence of significant atherosclerosis and iliac tortuosity in this patient population.

## Methods

The records of a prospectively maintained single-center aneurysm database collected by the senior author (RJT) were reviewed to identify 100 consecutive SAH patients. Femoral artery cannulation and placement of an arterial sheath were performed using Seldinger technique [3]. Using an unsubtracted oblique femoral arteriogram injected through the sheath, the presence of significant atherosclerosis, iliac tortuosity, and the CFA bifurcation were assessed.

**Figure 1:**
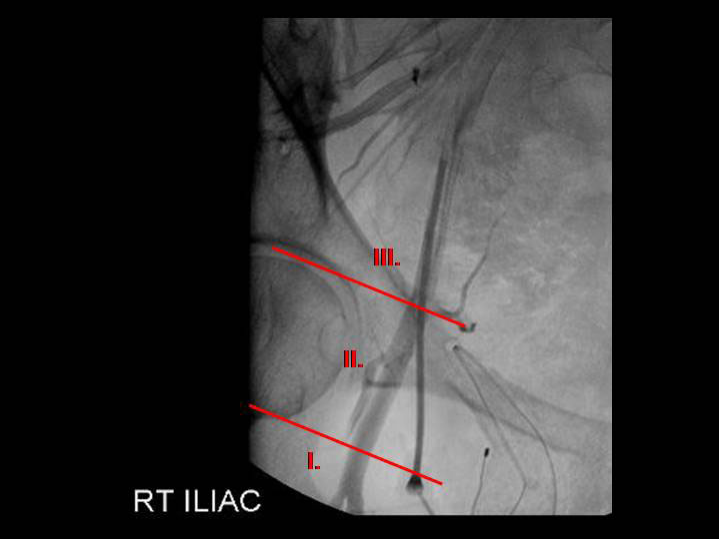
**Femoral Angiogram Demonstrating Grading System**. Two parallel lines drawn to the top and bottom of femoral head. A grade 1 bifurcation occurs below the femoral head. A grade 2 bifurcation occurs in the region of the femoral head. A grade 3 bifurcation occurs above the femoral head.

The CFA bifurcation was graded according to its position with respect to the femoral head. Two lines were drawn parallel to the top and to the bottom of the femoral head. A grade 1 bifurcation was defined as a bifurcation that occurred below the femoral head (Figure 1). A bifurcation that occurred between the boundaries of the upper and lower lines (in the region of the femoral) head was defined as grade 2. A grade 3 bifurcation was defined as a bifurcation that occurred above the femoral head. These data were compared to the demographic, radiographic, and clinical characteristics of these patients.

We also determined the presence of atherosclerosis as defined by an atherosclerotic plaque causing >50% stenosis within the external iliac/femoral system. Iliac tortuosity, defined by at least one coil of vessel within the external iliac/femoral system, was also recorded.

## Results

We found a grade 1 CFA bifurcation in 50 patients (50%, mean age 51.2 ± 15.8 years), grade 2 in 40 patients (40%, mean age 55.5 ± 14.8 years), and grade 3 in 10 patients (10%, mean age 58.2 ± 10.1 years). Whereas 30 of 90 patients with CFA grades I or II were male (33%), only 10% with grade 3 were male (1 of 10, p = 0.12). There were no significant differences in the demographics of the three groups (Table 1). Mean age for the presence of significant atherosclerosis was 65.5 +/- 11.2 years versus 50.9 +/- 14.5 years (p < 0.001) without. The mean age of iliac tortuosity was 64.9 +/- 11.4 years versus 50.3 +/- 14.4 years (p < 0.001) without.

**Table 1:** Baseline Demographics

		Grade 1 Bifurcation	Grade 2 Bifurcation	Grade 3 Bifurcation
Number of Patients (n)		50	40	10

Age (years, mean ± SD)		51.2 ± 15.8	55.5 ± 14.8	58.2 ± 10.1

Sex (Female (%))		72	65	90

Race	Caucasian (%)	66	55	20
	
	African American(%)	32	38	60
	
	Other (%)	2	1	20

Significant Atherosclerosis (%)		20	15	30

Iliac Tortuosity (%)		16	28	40

## Discussion

We sought to demonstrate the location of the CFA bifurcation to provide further insight into puncture placement in order to safely obtain arterial access in a population of patients presenting with SAH. The CFA bifurcation was in the region of the femoral head 40% of the time and below the femoral head 50% of the time. We found a high bifurcation of the CFA (above the femoral head) in 10% of our patient population. Of these 10 patients, only 1 was male (10%) compared to grade 1 or 2 bifurcations which had 33% males. The mean ages for atherosclerosis and iliac tortuosity were 65.5 years and 64.9 years, respectively.

There is wide spread agreement that the CFA is the optimal location for artery puncture. The inguinal skin crease, maximal pulsation and/or bony landmarks are most frequently used among angiographers. However, these landmarks are far from perfect. Angiographers using the inguinal skin crease punctured below the crease 47% of the time, above the crease 21% of the time and at the crease 26% of the time [4]. Studies have shown that the bifurcation of the CFA is 1-4 cm above the inguinal crease 77% of the time. In addition, common palpable landmarks may be obscured by obesity, scarring, hypotension in shock or prior hematoma [5].

In 1974, Grossman recommended using fluoroscopy over the femoral head to locate the CFA [6]. Garrett et al. showed that in their series of patients referred for elective cardiac catheterization the CFA bifurcated below the middle of the femoral head 99% of the time and bifurcated below the inferior border of the femoral head 80% of the time. In their series, the femoral artery never bifurcated above the inguinal ligament and bifurcated below the inguinal crease 22% of the time. They concluded that in their patient population, there was a reliable relationship of the femoral head which could be used to help localize the CFA [1].

This is in contrast to our results in which the CFA bifurcation was above the femoral head 10% of the time in patients presenting with SAH, which is much higher than the 1% seen by Garrett et al. The higher incidence of CFA bifurcation above the femoral head in patients presenting with SAH is an important technical element when performing arterial puncture and suggests that the benefit of utilizing the femoral head guided fluoroscopic technique may be diminished in this population. The surgeon should be aware of an increased risk of thrombosis and arteriovenous fistula formation if the puncture is distal into branch vessels.

The incidence of asymptomatic and symptomatic atherosclerotic disease increases in proportion with age. Regardless of the presence or absence of other risk factors, atherosclerotic disease is considered present to some degree in patients aged 65 or older [7]. Consistent with this, in our study population we found significant atherosclerosis and iliac tortuosity in patients over 65 years of age. The atherosclerotic disease of the CFA is usually in continuum with the atherosclerosis of the iliac (proximal) or femoro-popliteal (distal) segments [8]. The posterior wall of the CFA is commonly involved in atherosclerotic disease while the anterior wall is relatively spared due to differential wall stress [9].

## Conclusions

Although a requisite element of endovascular treatment in SAH patients, femoral access can be complicated by a high common femoral artery bifurcation and the presence of atherosclerotic disease and/or iliac artery tortuosity. In this study, we found a grade 3 CFA bifurcation in 10% patients, with 90% of these patients being female. We also found the presence of atherosclerotic disease and iliac tortuosity in patients older than 65 years of age.

## List of Abbreviations

(CFA): Common Femoral Artery; (SAH): Subarachnoid Hemorrhage.

## Competing interests

The authors declare that they have no competing interests.

## Authors' contributions

ARP drafted the manuscript and participated in the statistical analysis. GPC participated in the analysis of the data and preparation of the manuscript. MGR participated in the analysis of the radiographs. JH conceptualized the analysis and participated in the maintenance of the database. RJT created and oversaw the clinical database. ALC conceived the study and oversaw all elements of its completion. All authors read and approved the final manuscript.
